# Evaluation of CTX-M steady-state mRNA, mRNA half-life and protein production in various STs of *Escherichia coli*

**DOI:** 10.1093/jac/dkv388

**Published:** 2015-11-26

**Authors:** Chelsie N. Geyer, Randal C. Fowler, James R. Johnson, Brian Johnston, Scott J. Weissman, Peter Hawkey, Nancy D. Hanson

**Affiliations:** 1 Department of Medical Microbiology and Immunology, Center for Research in Anti-Infectives and Biotechnology, Creighton University School of Medicine, 2500 California Plaza, Omaha, NE 68178, USA; 2 Veterans Affairs Medical Center, Minneapolis, MN, USA; 3 University of Minnesota, Minneapolis, MN, USA; 4 Center for Global Infectious Diseases Research, Seattle Children's Research Institute, Seattle, WA 98101, USA; 5 Public Health England (PHE), West Midlands Public Health Laboratory, Heart of England NHS Foundation Trust, Bordesley Green East, Birmingham B9 5SS, UK; 6 Institute of Microbiology and Infection, School of Biosciences, School of Immunity and Infection, University of Birmingham, Edgbaston Campus, Birmingham B15 2TT, UK

## Abstract

**Objectives:**

High levels of β-lactamase production can impact treatment with a β-lactam/β-lactamase inhibitor combination. Goals of this study were to: (i) compare the mRNA and protein levels of CTX-M-15- and CTX-M-14-producing *Escherichia coli* from 18 different STs and 10 different phylotypes; (ii) evaluate the mRNA half-lives and establish a role for chromosomal- and/or plasmid-encoded factors; and (iii) evaluate the zones of inhibition for piperacillin/tazobactam and ceftolozane/tazobactam.

**Methods:**

Disc diffusion was used to establish zone size. RNA analysis was accomplished using real-time RT–PCR and CTX-M protein levels were evaluated by immunoblotting. Clinical isolates, transformants and transconjugants were used to evaluate mRNA half-lives.

**Results:**

mRNA levels of CTX-M-15 were up to 165-fold higher compared with CTX-M-14. CTX-M-15 protein levels were 2–48-fold less than their respective transcript levels, while CTX-M-14 protein production was comparable to the observed transcript levels. Nineteen of 25 *E. coli* (76%) had extended CTX-M-15 mRNA half-lives of 5–15 min and 16 (100%) CTX-M-14 isolates had mRNA half-lives of <2–3 min. Transformants had mRNA half-lives of <2 min for both CTX-M-type transcripts, while transconjugant mRNA half-lives corresponded to the half-life of the donor. Ceftolozane/tazobactam zone sizes were ≥19 mm, while piperacillin/tazobactam zone sizes were ≥17 mm.

**Conclusions:**

CTX-M-15 mRNA and protein production did not correlate. Neither *E. coli* ST nor phylotype influenced the variability observed for CTX-M-15 mRNA or protein produced. mRNA half-life is controlled by a plasmid-encoded factor and may influence mRNA transcript levels, but not protein levels.

## Introduction

In Gram-negative bacteria, β-lactamase production is the most common mechanism identified conferring resistance to β-lactams.^1^ Development of β-lactamase inhibitors such as clavulanic acid, sulbactam and tazobactam provides an effective method for evading this resistance mechanism. These inhibitors have minimal antibiotic activity against enteric bacilli when used alone; however, a synergistic effect is created when administered in combination with a penicillin or cephalosporin. Currently there are four penicillin/inhibitor combinations approved for clinical use in the USA, including ampicillin/sulbactam, amoxicillin/clavulanate, ticarcillin/clavulanate and piperacillin/tazobactam.^2^ Recently, the FDA approved the use of ceftolozane/tazobactam for the treatment of complicated urinary tract infections. By irreversibly binding to the enzyme, the β-lactamase inhibitor protects the β-lactam antibiotic from being hydrolysed by the β-lactamase. Such β-lactamase inhibitor combinations are highly active against most class A β-lactamases, but are poorly active against classes C and D, and inactive against class B β-lactamases.^2^ The clinical efficacy of a β-lactamase inhibitor/β-lactam combination depends on many factors, including concentration of inhibitor used in the formulation, amount of β-lactamase produced by the bacterial cell and the concentration of antibiotic that enters the periplasmic space. Emergence of resistance to β-lactam/β-lactamase inhibitor combinations can severely impact the ability to treat serious respiratory tract, urinary tract and bloodstream infections. Therefore, the ratio of β-lactam/β-lactamase inhibitor used in combinations is critical because use of an inappropriate amount of inhibitor may impact the therapeutic value of the drug.^3^

CTX-M-producing *Escherichia coli* are predominately the pandemic ST131 clone and frequently cause urinary tract infections.^4,5^ The rapid spread of these strains has led to the CTX-M pandemic.^6^ Two major genotypes of CTX-M have become established worldwide, CTX-M-15 and CTX-M-14. These CTX-M producers have contributed to both hospital- and community-acquired urinary tract infections.^6–11^ *E. coli* represents 50% of infections leading to uroseptic shock in hospitalized patients and the majority of uroseptic infections in these patients originate from the community.^12,13^ β-Lactam/β-lactamase inhibitor combinations can be an effective treatment for infections caused by CTX-M-producing organisms.^2,6,10,13,14^

Recently, our laboratory has documented elevated levels of CTX-M-15 mRNA, in comparison with CTX-M-14 mRNA levels, in *E. coli* from human urine samples.^15^ This difference in steady-state mRNA expression between CTX-M-15 and CTX-M-14 producers was observed from isolates collected from various geographical locations worldwide indicating that this observation was not due to a local clonal population of isolates (Table 1). Steady-state mRNA levels take into account both the rates of mRNA synthesis and degradation (mRNA half-life).^16^ Thus, the observed differences in expression levels between *bla*_CTX-M-14_ and *bla*_CTX-M-15_ could be due to an extended mRNA half-life for *bla*_CTX-M-15_ transcripts compared with *bla*_CTX-M-14_ transcripts. It is possible that elevated levels of CTX-M-15 transcripts could lead to elevated levels of CTX-M-15 protein production negating the effect of β-lactamase inhibitors. The goal of this study was to compare the mRNA expression and protein levels of CTX-M-14 and CTX-M-15 β-lactamases among clinical *E. coli* isolates of varying STs and phylotypes from human urine samples. In addition, we evaluated the mRNA half-life of these transcripts and the susceptibility of these clinical isolates to piperacillin/tazobactam in addition to evaluating the zone of inhibition for ceftolozane/tazobactam.^17^

**Table 1.** Characteristics, expression data and susceptibility data (zone sizes in mm) for CTX-M-15- and CTX-M-14-producing *E. coli* isolates used in this study

Strain	Geographical location of isolation	*bla*_CTX-M_ allele	ST	Phylotype	Relative fold change in expression ± SD	Relative fold change in protein ± SD	Zone diameter (mm)	
							piperacillin/ tazobactam	ceftolozane/ tazobactam
D14	Omaha, NE	CTX-M-14	ST405	D2	1	1 ± 0.15	22	21
CUMC 247	Omaha, NE	CTX-M-15	ST131	B2	72 ± 12.56	2.5 ± 0.39	23	21
XQ12	Seattle, WA	CTX-M-15	ST131	B2	21 ± 5.12	10.3 ± 3.6	22	22
XQ35	Seattle, WA	CTX-M-15	ST131	B2	8 ± 0.87	1.06 ± 0.87	20	25
A15	Omaha, NE	CTX-M-15	ST44	D2	15 ± 1.28	ND		
C15	Omaha, NE	CTX-M-15	ST405	B1	48 ± 0.70	3.9 ± 2	23	22
H15	Omaha, NE	CTX-M-15	ST205	B2	20 ± 0.68	12.2 ± 5.0	17	23
W15	Omaha, NE	CTX-M-15	ST131	B2	29 ± 1.43	2.6 ± 0.25	17	21
FHM6	India	CTX-M-15	ST131	B2	48 ± 8.41	3.2 ± 1.21	21	19
RS059	UK	CTX-M-15	ST131	B2	77 ± 32.47	10.0 ± 2.43	23	20
RS061	UK	CTX-M-15	ST131	B2	52 ± 3.65	7.7 ± 2.33	24	20
RS007	UK	CTX-M-15	ST131	B2	1 ± 0.09	ND		
RS120	UK	CTX-M-15	ST131	B2	1 ± 0.14	ND		
F010	UK	CTX-M-15	ST69	D1	26 ± 2.51	3.5 ± 1.5	20	25
F024	UK	CTX-M-15	ST2076	D1	46 ± 2.30	ND		
F076	UK	CTX-M-15	ST182	D1	74 ± 6.92	ND		
RS135	UK	CTX-M-15	ST131	B2	165 ± 27.76	22.32 ± 10.7	18	20
RS153	UK	CTX-M-15	ST617	A2	37 ± 2.75	8.1 ± 1.27	25	30
CMB106	Minneapolis, MN	CTX-M-15	ST131	B2	51 ± 8.17	2.1 ± 0.46		
JJ2052 S	Evanston, IL	CTX-M-15	ST90	B1	32 ± 1.58	2.5 ± 1.97		
JJ2052 F	Evanston, IL	CTX-M-15	ST90	B1	32 ± 3.90	2.4 ± 1.80		
JJ2053	Evanston, IL	CTX-M-15	ST131	B2	−14 ± 0.04^a^	UD		
JJ2131	Minneapolis, MN	CTX-M-15	ST167	A2	47 ± 10.83	2.9 ± 1.79		
JJ2235 S	Houston, TX	CTX-M-15	ST167	A2	39 ± 11.27	15.4 ± 2.75		
JJ2235 F	Houston, TX	CTX-M-15	ST167	A2	62 ± 17.32	4.4 ± 2.41		
JJ2236	New York, NY	CTX-M-15	ST648	D2	65 ± 10.88	14.1 ± 4.86		
JJ2241	New Brunswick, NJ	CTX-M-15	ST131	B2	14 ± 3.97	−2 ± 0.10^a^		
JJ2242	Rochester, NY	CTX-M-15	ST131	B2	6 ± 0.44	UD		
JJ2243	Milwaukee, WI	CTX-M-15	ST131	B2	1 ± 0.90	UD		
JJ2244	Detroit, MI	CTX-M-15	ST131	B2	29 ± 4.22	1.7 ± 0.62		
JJ2246 S	Ewa Beach, HI	CTX-M-15	ST410	A2	60 ± 15.83	5.1 ± 1.50		
JJ2246 F	Ewa Beach, HI	CTX-M-15	ST410	A2	155 ± 41.89	28.1 ± 2.48	20	22
JJ2247	Galveston, TX	CTX-M-15	ST131	B2	48 ± 7.02	−1.3 ± 0.42^a^	20	22
JJ2251	New York, NY	CTX-M-15	ST131	B2	49 ± 5.06	1.6 ± 0.93	20	26
JJ2253 SW	Minneapolis, MN	CTX-M-15	ST410	A2	36 ± 5.94	2.4 ± 0.57		
JJ2253 RG	Minneapolis, MN	CTX-M-15	ST410	A2	34 ± 5.91	12.1 ± 3.10		
JJ2267	Salt Lake City, UT	CTX-M-15	ST648	D2	42 ± 25.83	9.5 ± 1.50		
JJ2431	New York, NY	CTX-M-15	ST131-like	B2	39 ± 9.42	23.8 ± 3.15		
MHHBS4	Spain	CTX-M-15	ST131	B2	49 ± 2.40	6.2 ± 2.10		
MHVlab2	France	CTX-M-15	ST131	B2	59 ± 15.05	16.1 ± 3.49		
QU015	Queensland, Australia	CTX-M-15	ST131	B2	14 ± 3.85	1.9 ± 0.55		
C14	Omaha, NE	CTX-M-14	ST648	D2	5 ± 2.08	5.0 ± 0.82	21	21
La14	Omaha, NE	CTX-M-14	ST648	D2	4 ± 1.68	6.2 ± 0.10	26	28
Lo14	Omaha, NE	CTX-M-14	ST405	D2	5 ± 0.39	3.1 ± .81		
N14	Omaha, NE	CTX-M-14	ST3856	A2	3 ± 0.40	6.2 ± 3.3		
F044	UK	CTX-M-14	ST131	B2	1 ± 0.15	−1.4 ± 0.09^a^	25	24
NL217	UK	CTX-M-14	ST131	B2	1 ± 0.19	1.6 ± 0.75	26	23
XQ10	Seattle, WA	CTX-M-14	ST38	D	1 ± 0.40	ND		
XQ13	Seattle, WA	CTX-M-14	ST68	D	2 ± 0.72	1.5 ± 0.90	23	21
XQ22	Seattle, WA	CTX-M-14	ST156	B2	1 ± 0.73	ND	23	23
XQ24	Seattle, WA	CTX-M-14	ST10	A	1 ± 0.17	ND	22	23
JJ2339	Ewa Beach, HI	CTX-M-14	ST38	D2	2 ± 0.64	2.1 ± 0.66		
JJ2354	Orange County, CA	CTX-M-14	ST354	D1	2 ± 0.76	1.4 ± 0.47		
JJ2356	Seattle, WA	CTX-M-14	ST46	A1	1 ± 0.26	3.7 ± 1.17		
FS-ESBL013	Denmark	CTX-M-14	ST10	D2	2 ± 0.53	1.3 ± 0.42		
FS-ESBL014	Denmark	CTX-M-14	ST38	A2	3 ± 1.55	13.9 ± 4.32		
FS-ESBL062	Denmark	CTX-M-14	ST10	D1	2 ± 0.47	11 ± 1.06		

ND, not determined; UD, undetected.

All transcript levels are relative to D14, which was used as the comparator and set to 1.

Fold changes in protein are normalized values.

^a^Down-regulation of gene or protein production.

## Methods

### Bacterial isolates and susceptibility testing

The study population comprised 57 CTX-M-producing *E. coli* clinical isolates chosen from 18 geographic locations to ensure that the data did not represent a local point-source clonal outbreak (Table 1). In addition to harbouring a CTX-M-14 or CTX-M-15 β-lactamase, most isolates possessed other β-lactamases such as TEM-1-like or OXA-1, as determined by family-specific PCR.^18^ Twenty-five CTX-M-15-producing and 16 CTX-M-14-producing *E. coli* were used to evaluate mRNA half-life. The donor strains used in conjugation studies included XQ12 (CTX-M-15), XQ35 (CTX-M-15), C15 (CTX-M-15), D14 (CTX-M-14) and XQ10 (CTX-M-14). J53 (sodium azide resistant) *E. coli* served as the first recipient strain in the conjugation strategy and SalLT2 (*Salmonella enterica* serovar Typhimurium LT2) was the intermediate recipient. The final recipient strains used for conjugation included J53 *E. coli*, K12 MG1655 (WT *E. coli*) and FHM16 (ST131 *E. coli* without a CTX-M enzyme). Vectors used for cloning and construction of transformants included pCR^®^2.1, pJET1.2/blunt, pSP-*luc*+, pUCP26, pACYC184 (chloramphenicol promoter intact), pMDR009 (chloramphenicol promoter deleted) and pMP220 (Table S1, available as Supplementary data at *JAC* Online). Disc diffusion assays were performed as previously described and piperacillin/tazobactam susceptibilities were interpreted using CLSI guidelines.^19^ In addition, zone sizes were determined for ceftolozane/tazobactam against 129 total isolates. No disc diffusion breakpoints for Enterobacteriaceae are currently available for ceftolozane/tazobactam; therefore, only zone sizes can be reported.

### Conjugation assays and clinical plasmid isolation

Conjugation studies were performed using a combination of broth and filter mating. Transconjugants were created to evaluate the possibility that a factor(s) encoded on the clinical plasmid was influencing the differential expression of CTX-M-14 and CTX-M-15 mRNA. Donor strains, XQ12 (CTX-M-15), XQ35 (CTX-M-15), C15 (CTX-M-15), D14 (CTX-M-14) and XQ10 (CTX-M-14) were conjugated with J53 *E. coli* using brain heart infusion (Difco, Thermo Scientific) broth mating. A donor to recipient ratio of 1: 2.5 was used in all conjugation experiments. Transconjugants were selected on LB agar with sodium azide (NaN_3_) 100 mg/L and cefotaxime 24 mg/L. J53 transconjugants were mated with SalLT2 and plated on MacConkey agar with cefotaxime at 24 mg/L. Following transfer into SalLT2, the CTX-M-harbouring plasmid was moved by filter mating into MG1655 and FHM16 with transconjugants selected using MacConkey agar supplemented with 24 mg/L cefotaxime. Plasmid transfer was confirmed via agarose gel electrophoresis (data not shown).^20^

### Cloning and sequencing

Transformants were constructed by amplifying the *bla*_CTX-M-14_ and *bla*_CTX-M-15_ structural genes and promoter regions by PCR, ligation of the PCR products into vector MDR009 (chloramphenicol promoter removed) and transformation into MG1655, J53 and FHM16 *E. coli*. The structural genes encoding CTX-M-14 and CTX-M-15 were sequenced from various clinical isolates along with their upstream promoter regions using primers listed in Table S2. All clones and subclones were sequenced. To create the CTX-M MG1655 transformants, pMRD009 was the final destination vector. For the luciferase clones, the CTX-M promoter regions were fused to the luciferase gene and cloned into vector pMP220, while CTX-M genes driven by the chloramphenicol or *lacZ* promoter were cloned into pACYC184 (ATCC) and pUCP26, respectively.

### Primer efficiency evaluations and determination of copy number using PCR

Because different primer sets were used for expression of CTX-M-14 and CTX-M-15, comparisons of the data obtained using these primers could not be made unless the primers amplified with the same efficiency. Total DNA was extracted using the Qiagen DNeasy^®^ Blood and Tissue kit. Ten-fold serial dilutions were prepared ranging from 250 ng to 0.025 ng. The master mix included 1× Rotor-Gene SYBR Green buffer (Qiagen, Valencia, CA, USA), 10 pmol of each forward and reverse CTX-M-specific real-time primer and RNase-free H_2_O for a total reaction volume of 40.5 μL. A separate master mix was prepared using the endogenous control primer set (*frr* or 16S rRNA) and these genes were used to normalize the expression data. A standard curve was constructed for both the CTX-M and endogenous control primer sets for each isolate. The *C*_t_ values were plotted against the logarithm of the DNA concentration. Each standard curve was generated from a linear regression of the plotted points. Using the slope of each standard curve, the PCR amplification efficiency (*E*) was calculated using the equation *E* = 10^−1/slope^–1,^21^ while the efficiency percentage was calculated using the equation *E* = (10^−1/slope^–1) × 100. The *R*^2^ value ranged from 0.97 to 0.99. The target [CTX-M-15, CTX-M-14 or luciferase (*luc*)] and reference (*frr*, 16S rRNA) primer sets did not vary more than 10% from each other.

### bla_CTX-M-15_ and bla_CTX-M-14_ relative copy number determination

Relative gene copy number quantification was calculated using the equation of Skulj *et al*.:^22^ copy number = (*Ec* average) *C*_t_^c^/(*Ep* average) *C*_t_^p^, where *E* is the primer efficiency, *c* is the normalization gene and *p* is the gene of interest. Copy number studies were completed on select CTX-M-14- and CTX-M-15-producing clinical isolates in Tables 2 and 3.

**Table 2.** Upstream region and mRNA half-life of selected strains of CTX-M-15 and CTX-M-14 producers

Strain	*bla*_CTX-M_ allele	Upstream region	mRNA half-life (min)
XQ12	CTX-M-15	IS*Ecp1*^a^	8 or 10^c^
C15	CTX-M-15	IS*Ecp1*^a^	5
XQ35	CTX-M-15	IS*Ecp1*^a^	<2
JJ2053	CTX-M-15	IS*Ecp1*^a^	9
JJ2241	CTX-M-15	IS*Ecp1*-like^b^	8
JJ2242	CTX-M-15	IS*Ecp1*-like^b^	<2
JJ2243	CTX-M-15	IS*Ecp1*-like^b^	12
JJ2244	CTX-M-15	IS*Ecp1*-like^b^	15
JJ2247	CTX-M-15	IS*Ecp1*-like^b^	2
JJ2131	CTX-M-15	IS*Ecp1*-like^b^	<2
JJ2235 S	CTX-M-15	IS*Ecp1*-like^b^	<2
JJ2235 F	CTX-M-15	IS*Ecp1*-like^b^	<2
JJ2236	CTX-M-15	IS*Ecp1*-like^b^	10
JJ2253 RG	CTX-M-15	ND	11
MHHBS4	CTX-M-15	non-IS*Ecp1*^b^	<2, 7 or 12^c^
MHVlab2	CTX-M-15	non-IS*Ecp1*^b^	9
QU015	CTX-M-15	non-IS*Ecp1*^b^	<2
JJ2251	CTX-M-15	non-IS*Ecp1*^b^	9
JJ2052 S	CTX-M-15	non-IS*Ecp1*^b^	2
JJ2052 F	CTX-M-15	non-IS*Ecp1*^b^	9
JJ2246 S	CTX-M-15	non-IS*Ecp1*^b^	<2 or 7^c^
JJ2246 F	CTX-M-15	non-IS*Ecp1*^b^	7
JJ2267	CTX-M-15	ND	9
JJ2253 SW	CTX-M-15	ND	5
JJ2243	CTX-M-15	ND	<2
D14	CTX-M-14	IS*Ecp1*^a^	<2
XQ10	CTX-M-14	IS*Ecp1*^a^	<2
C14	CTX-M-14	IS*Ecp1*^a^	<2
La14	CTX-M-14	IS*Ecp1*^a^	<2
Lo14	CTX-M-14	IS*Ecp1*^a^	3
N14	CTX-M-14	IS*Ecp1*^a^	<2
F044	CTX-M-14	IS*Ecp1*^a^	<2
XQ22	CTX-M-14	IS*Ecp1*^a^	<2
XQ24	CTX-M-14	IS*Ecp1*^a^	<2
FS-ESBL014	CTX-M-14	ND	<2
JJ2339	CTX-M-14	ND	<2
JJ2354	CTX-M-14	ND	<2
JJ2356	CTX-M-14	ND	<2
NL217	CTX-M-14	ND	<2
FS-ESBL013	CTX-M-14	ND	3
FS-ESBL062	CTX-M-14	ND	3

Each mRNA half-life represents one experiment. Studies were not completed in triplicate due to cost.

Half-life calculated using the 2001 method of Pfaffl.^26^

^a^Confirmed by sequence analysis.

^b^Upstream region PCR mapped.

^c^Indicates the graph intersects the 50% transcript remaining line at more than one timepoint.

**Table 3.** Steady-state mRNA expression and mRNA half-lives for CTX-M-14 and CTX-M-15 transconjugants in different *E. coli* backgrounds

Clinical isolate donor/ transconjugant	*bla*_CTX-M_ allele	Relative mRNA levels ± SD	mRNA half-life (min)^a^
XQ12	CTX-M-15	20 ± 5.12	8–10
XQ12-J53	CTX-M-15	23 ± 10.58	7
XQ12-K12 MG1655	CTX-M-15	31 ± 4.41	9
XQ12-FHM16	CTX-M-15	26 ± 3.63	9
XQ35	CTX-M-15	4 ± 1.60	<2
XQ35-J53	CTX-M-15	7 ± 3.47	<2
XQ35-K12 MG1655	CTX-M-15	26 ± 7.21	<2
XQ35-FHM16	CTX-M-15	16 ± 3.12	<2
C15	CTX-M-15	56 ± 17.52	5
C15-J53	CTX-M-15	62 ± 6.89	5
C15-K12 MG1655	CTX-M-15	25 ± 12.73	5
C15-FHM16	CTX-M-15	59 ± 28.98	5 or 11
D14	CTX-M-14	1 ± 0.20	<2
D14-J53	CTX-M-14	1 ± 0.46	<2
XQ10	CTX-M-14	0.73 ± 0.48	<2
XQ10-J53	CTX-M-14	0.76 ± 0.39	<2

All fold changes are relative to D14.

^a^Calculated using the 2001 method of Pfaffl.^26^

### MLST and phylotyping

Seven-locus MLST was done according to the Achtman system (http://mlst.ucc.ie/mlst/dbs/Ecoli). Major *E. coli* phylogenetic groups (A, B1, B2 or D) were determined using an established triplex PCR method.^23^

### RNA isolation and mRNA expression assays

RNA was isolated using TRIzol^®^ Max^TM^ (Invitrogen) from 1.5 mL of mid-logarithmic phase culture grown in Mueller–Hinton broth (OD_600_ of ∼0.5).^24^ Genomic DNA was removed by RQ1 DNase (Promega, Madison, WI, USA) treatment. 250 ng of DNA-free RNA was used in 50 μL PCRs that consisted of the 1× concentration of QuantiTect^®^ SYBR^®^ Green RT–PCR master mix, RT mix (Qiagen, Hilden, Germany) and 25 pmol of each primer. RT–PCR was performed on the Rotor Gene Q 5plex high resolution melt system (Qiagen, Valencia, CA, USA) that included RT activation at 50°C for 40 min, HotStart Taq DNA polymerase activation at 95°C for 15 min and three step cycling conditions for 40 cycles. Cycling parameters included denaturation at 95°C for 30 s, annealing at 51°C for 30 s and extension at 72°C for 30 s. Relative mRNA expression for CTX-M-14 and CTX-M-15 was calculated using the 2^−ΔΔCt^ method with D14 as the comparator.^24,25^ The single copy gene, *frr* was used to normalize the data. Three independent RNA isolations and three individual RT–PCR assays were completed with a coefficient of variation of <10%.

### Evaluation of mRNA half-lives

Cultures were grown to mid-logarithmic phase (OD_600_ of ∼0.5) in Mueller–Hinton broth and treated with 200 mg/L rifampicin (Sigma Aldrich, St Louis, MO, USA). Cells were harvested by centrifugation at 0, 2, 4, 6, 8, 10, 15, 20, 25 and 30 min post-rifampicin addition and RNA was isolated using TRIzol^®^ Max^TM^. Real-time RT–PCRs were done using 250 ng of DNA-free RNA. Data were normalized to the 16S rRNA gene of *E. coli*. The equation by Pfaffl^26^ was used to determine the ratio of transcript that remained at each timepoint.

### Immunoblot for CTX-M-14/CTX-M-15 detection

The linear response range of the Stain-Free fluorescence and the anti-CTX-M antibody for bacterial lysates was determined by performing a western blot on a dilution series of total protein ranging from 40 to 0.625 μg as previously described.^27^ The protein linearity ranged from 5 to 40 μg (Figure S1) and the linear range of the anti-CTX-M antibody was from 2.5 to 40 μg (Figure S2). Therefore, 10 μg of total protein and an antibody dilution factor of 1: 45 000 were used for immunoblot analyses.

Whole cell protein lysate was prepared from clinical isolates listed in Table 1 as previously described.^28^ Stain-Free SDS-PAGE, imaging and total protein normalization were carried out as previously described.^27^ A custom polyclonal antibody specific for CTX-M-14 and CTX-M-15, directed toward the peptide sequence CAIPGDPRDTT was generated by GenScript (Piscataway, NJ, USA) and the secondary antibody (horseradish peroxidase-goat anti-rabbit IgG) was used with a dilution factor of 1: 50 000. The chemiluminescent signal intensity of CTX-M was normalized to the Stain-Free fluorescent signal intensity of total protein for each isolate. K12 MG1655 (WT *E. coli*) and FHM16 (clinical isolate without CTX-M) lysates were used as controls for cross-reactivity to proteins other than CTX-M. To ensure that the antibody recognized each protein with the same affinity, *E. coli* transformants expressing each CTX-M gene (*bla*_CTX-M-15_ and *bla*_CTX-M-14_) from a common plasmid driven by the same promoter element were used to compare the efficiency of antibody detection (data not shown). Three biological replicates of each isolate were independently collected and the means of the normalized data sets were used to calculate the relative amount of CTX-M production.^29^ Statistical significance of CTX-M protein levels between isolates was evaluated using a *t*-test (double-sided and paired) performed with GraphPad Prism software, version 4.0.

## Results

### Relative levels of bla_CTX-M-14_ and bla_CTX-M-15_ transcripts

The CTX-M β-lactamases originated from the *Kluyvera* species, a group of environmental, non-pathogenic organisms that contain chromosomally encoded CTX-M β-lactamase genes.^30^ Therefore, to evaluate the relative expression of both mRNA and protein levels among the *E. coli* CTX-M-producing isolates, an *E. coli* isolate (D14) that produced a low amount of CTX-M transcript compared with the other study isolates was used as the comparator (Table 1). mRNA analysis of CTX-M-specific transcripts relative to isolate D14 showed that *E. coli* isolates expressing CTX-M-15 had mRNA transcript levels that ranged from 14-fold lower to 165-fold higher than the comparator. However, most CTX-M-15 producers (32 of 40; 80%) showed 20–165-fold increases in *bla*_CTX-M-15_ transcripts. When mRNA levels of the CTX-M-14 producers were compared with each other relative to the comparator strain, D14, the range in expression among these isolates was only 5-fold (Table 1). Among all the isolates evaluated, the trend observed for the elevated expression of CTX-M-15 transcripts and the lower level of CTX-M-14 transcripts were consistent among the isolates regardless of the geographic location from which the isolates were collected.

### Relationship between CTX-M mRNA expression and E. coli ST and phylotype

Various STs and phylotypes were evaluated for CTX-M gene expression and protein production. No correlation was observed with respect to specific ST and/or phylotype of *E. coli* expressing CTX-M-14 or CTX-M-15 genes (Table 1). For example, strains RS135 and JJ2053 were both identified as ST131 phylotype B2 isolates. However, RS135 expressed the *bla*_CTX-15_ gene 165-fold higher than the comparator D14 strain whereas strain JJ2053 expressed the *bla*_CTX-15_ gene 14-fold less than the comparator. Twenty-one of 41 isolates were ST131 phylotype B2 with *bla*_CTX-15_ gene expression ranging from a 14-fold decrease to a 165-fold increase in CTX-M-15 mRNA levels relative to the comparator strain, D14. Three ST405 isolates were evaluated for CTX-M gene expression. Two strains expressed CTX-M-14 and were both phylotype D2. Strain Lo14 expressed the *bla*_CTX-14_ gene 5-fold higher than the comparator strain, D14. The CTX-M-15-producing ST405 strain was of phylotype B1 and expressed *bla*_CTX-M-15_ 48-fold higher than the comparator, D14. Four strains were phylotype D1, but were all different STs (ST69, ST2076, ST183 and ST354). ST354 expressed the *bla*_CTX-M-14_ gene only 2-fold higher than the *bla*_CTX-14_ mRNA expression of D14, but the *bla*_CTX-M-15_ mRNA expression observed in the D1 phylotypes ranged from 26- to 74-fold higher compared with D14.

### Determination of CTX-M-14/CTX-M-15 mRNA half-lives from clinical isolates

The differential expression of mRNA was not attributed to the ST or the phylotype of the organism. Therefore, we wanted to determine whether the differential expression between these two types of CTX-M transcripts was occurring at the initiation of transcription or post-transcriptionally due to mRNA stability differences. The promoter regions of 29 strains encoding CTX-M-14 or CTX-M-15 were sequenced and most of the strains had identical upstream sequences that housed the insertion sequence IS*Ecp*1 (Table 2). When PCR mapping data were used to define the promoter, there was no difference in the trends of CTX-M-15 expression for IS*Ecp*1-like upstream elements versus those strains that did not generate a specific amplicon for the IS*Ecp*1 element (for example, strains JJ2236 and JJ2246F; Table 2). Taken together, these data indicated that transcription initiation was probably not the cause of the differences observed in the mRNA levels.

mRNA half-life, i.e. the degradation rate of a transcript, can impact steady-state mRNA levels. Therefore, mRNA half-life studies were performed. The average half-life of an *E. coli* transcript is ∼2–3 min.^16^ Sixteen CTX-M-14-producing clinical *E. coli* isolates were evaluated for mRNA half-life. Each isolate had a half-life of ≤3 min (Table 2 and Figures S3–S7). However, 16 of 25 CTX-M-15-producing *E. coli* isolates had an extended half-life of between 5 and 15 min. The remaining nine CTX-M-15 clinical *E. coli* isolates had mRNA half-lives of ≤2 min. The difference in mRNA half-life observed for these isolates could be the result of chromosomal- or plasmid-encoded factors.

### Factors controlling CTX-M mRNA half-life: chromosomal or plasmid encoded

To determine the contribution of chromosomal- or plasmid-encoded factors to the observed differences in the half-lives of the *bla*_CTX-M_ transcripts, candidate strains were chosen and transconjugants and transformants constructed (Table 3). DNA from isolates D14 and XQ12 were used as a template to construct the transformants. For the CTX-M-15-producing isolates, three clinical isolates representing three distinct mRNA half-lives were selected to be conjugation donors and included XQ12 (half-life of 8–10 min), XQ35 (half-life of <2 min) and C15 (half-life of 5 min). Three different recipient strains were used for the conjugations for the CTX-M-15-containing plasmid: J53, MG1655 and FHM16 (Table 3). For the CTX-M-14-producing isolates, two clinical isolates both having an mRNA half-life of <2 min were selected for conjugation studies and included D14 and XQ10.

Before the half-lives of the CTX-M transconjugant transcripts were determined, the gene copy number and CTX-M steady-state expression were evaluated. CTX-M gene copy number was determined for the D14, XQ10 and XQ12 transconjugants. The copy number of the CTX-M genes for the transconjugants tested was identical to the gene copy number determined for the clinical donor strains, which was 1. D14 was used as the comparator strain when evaluating steady-state expression of the transconjugants to remain consistent with the initial expression data (Table 3). For most of the transconjugants, the mRNA expression levels were similar to those of the clinical isolate from which they were created (Table 3). Compared with D14, CTX-M-15 mRNA expression in the XQ12 clinical isolate was 20-fold higher and in the XQ12-J53, XQ12-MG1655 and XQ12-FHM16 transconjugants was comparable at 23-, 31- and 26-fold higher (Table 3). The steady-state mRNA expression level for the XQ35 clinical isolate was 4-fold higher than the comparator strain D14 and expression in the J53, MG1655 and FHM16 transconjugants was 7-, 26- and 16-fold higher, respectively (Table 3). Interestingly, the mRNA expression level of the XQ35-MG1655 and XQ35-FHM16 transconjugants was ∼2- to 6-fold higher compared with the XQ35 clinical isolate and the J53 transconjugant. Steady-state mRNA expression levels for the C15 clinical isolate and its J53, MG1655 and FHM16 transconjugants were 56-, 62-, 25- and 59-fold higher, respectively when compared with D14. The CTX-M-15 expression level from the C15-MG1655 transconjugant was 2–3-fold lower compared with the J53 and FHM16 transconjugants (Table 3). The CTX-M-14 steady-state mRNA levels for XQ10 and D14 and their J53 transconjugants were both similar to those of the D14 clinical isolate (Table 3).

Variation in steady-state levels of mRNA expression was observed between some of the CTX-M transconjugants, but not all, when compared with the clinical isolate. These differences were not due to gene copy number (all isolates had one copy of the gene), but could be attributed to transcription initiation events or transcript half-life in these different genetic backgrounds. To test the influence of these genetic backgrounds on mRNA stability, the mRNA half-life of each of the transcripts expressed in the transconjugants was measured (Table 3 and Figures S3–S7). Despite the difference in genetic background (J53 versus MG1655 versus ST131), each of the transconjugant's mRNA half-lives reflected the mRNA half-life of the clinical isolate from which the plasmid was obtained. Specifically, for transconjugant XQ12 the CTX-M-15 transcript had an extended half-life of 7–9 min, the XQ35 transconjugant had a *bla*_CTX-M-15_ mRNA half-life of <2 min and the C15 transconjugant had a *bla*_CTX-M-15_ half-life of 5 min (Table 3). The same trend was seen with the CTX-M-14 transconjugants, each of which had a *bla*_CTX-M-14_ half-life of <2 min, similar to the parent strain. These data indicated that plasmid-encoded factors and not chromosomally encoded factors were contributing to the difference in mRNA half-life observed between *bla*_CTX-M-14_ and *bla*_CTX-M-15_.

To verify further that a clinical plasmid-encoded factor(s) was responsible for mRNA stability, the mRNA half-lives of the CTX-M-14 and CTX-M-15 K12 transformants were evaluated. To create these constructs, the structural CTX-M-14 or CTX-M-15 gene and upstream promoter regions were amplified, cloned into pMDR009 and transformed into K12 MG1655 or FHM16 (ST131).^31^ When mRNA half-lives were evaluated in the MG1655 transformants, both the CTX-M-14 and CTX-M-15 transcripts were <2 min (Figure S7). To determine whether the ST131 genetic background would influence the CTX-M-15 transcript half-life, the mRNA was evaluated in the transformant of ST131 *E. coli* strain, FHM16. The mRNA half-life of the CTX-M-15 transcript in this transformant was also <2 min (Figure S7). These data substantiated that a plasmid-encoded factor(s), rather than the chromosomal background was responsible for the extended half-lives of the CTX-M-15 transcripts.

To rule out any contribution of the CTX-M native promoters or the structural genes themselves on CTX-M half-life, the half-lives of CTX-M promoter/luciferase fusions, *lacZ* promoter/CTX-M clones and chloramphenicol promoter/CTX-M clones were evaluated. When the promoter regions of the CTX-M-14 and CTX-M-15 genes were fused to luciferase, the half-life of the luciferase gene was <2 min for all clones (data not shown). Furthermore, the half-lives of the CTX-M-14 and CTX-M-15 transcripts driven by heterologous promoters (*lacZ* or chloramphenicol) were <2 min (data not shown). All clones discussed above included either the CTX-M promoter or structural gene, which had been removed from its native clinical plasmid and transformed into a K12 *E. coli* host.

### Relative levels of CTX-M-14 and CTX-M-15 protein

The elevated levels of the CTX-M-15 mRNA transcripts suggested that the level of CTX-M-15 enzyme produced by those *E. coli* isolates would also be elevated, perhaps jeopardizing the effectiveness of β-lactam/β-lactamase inhibitor combinations. Therefore, western blots were used to evaluate the relative level of CTX-M β-lactamase produced by the study isolates listed in Table 1.

**Figure 1.**
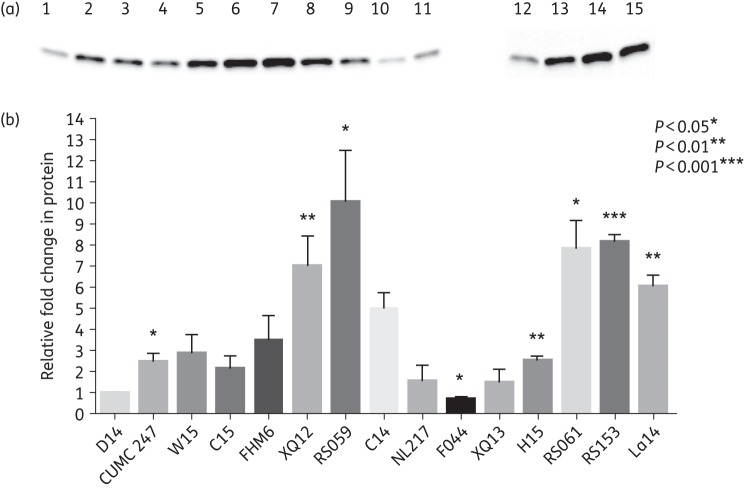
(a) Immunoblots for CTX-M-14 and CTX-M-15 from 15 representative clinical isolates. Protein levels were compared and analysed relative to *E. coli strain* D14. Lane 1, D14 (CTX-M-14); lane 2, CUMC 247 (CTX-M-15); lane 3, W15 (CTX-M 15); lane 4, C15 (CTX-M-15); lane 5, FHM6 (CTX-M-15); lane 6, XQ12 (CTX-M-15); lane 7, RS059 (CTX-M-15); lane 8, C14 (CTX-M-14); lane 9, NL217 (CTX-M-14); lane 10, F044 (CTX-M-14); lane 11, XQ13 (CTX-M-14; lane 12, H15 (CTX-M-15); lane 13, RS061 (CTX-M-15); lane 14, RS153 (CTX-M-15); lane 15, La14 (CTX-M-14). (b) Relative fold changes in CTX-M-15 and CTX-M-14 protein from the 15 isolates from (a) normalized to total protein using Stain-Free technology. All protein levels are relative to D14. Statistical significance of the isolates relative to D14 was evaluated using a *t*-test (two-tailed and paired) performed with GraphPad Prism 6.0.

An ∼1: 1 relationship between mRNA and protein production was observed for the CTX-M-14-producing isolates (Table 1 and Figure 1a and b). For example, *E. coli* isolate C14 expressed both its CTX-M-14 mRNA and protein at levels 5 times higher than the comparator isolate, D14. This trend in mRNA and protein production was seen with all of the CTX-M-14-producing isolates with levels ranging between 1- and 6-fold higher compared with isolate D14 (Table 1). In contrast, this direct relationship was not observed for CTX-M-15-producing isolates. While mRNA levels for the CTX-M-15 transcript ranged from 14-fold lower to 165-fold higher than the CTX-M-14 mRNA levels of isolate D14, the corresponding protein levels ranged from undetected to 10-fold higher (Table 1 and Figure 1a and b). In addition, the higher levels of CTX-M-15 mRNA did not always correlate with the highest levels of CTX-M-15 protein production. For example, isolate RS153 had an mRNA level 37-fold higher than the comparator strain, D14, but its protein level was only 8-fold higher. While a 77-fold increase in mRNA levels for strain RS059 was observed, only a 10-fold increase in protein production was noted. Isolate C15 had a 48-fold increase in mRNA compared with only a 2-fold increase in protein. Moreover, isolates JJ2246F and RS135 had the most pronounced increases for both mRNA and protein production of CTX-M-15 with a 155-fold and 165-fold increase in mRNA and a 28-fold and 22-fold increase in protein, respectively.

### Susceptibility to β-lactam/β-lactamase inhibitor combinations

Protein production among the test isolates ranged from no detectable level of protein observed to a 28-fold increase in protein production for strain JJ2246F. The level of β-lactamase production is a key contributor to the β-lactam-resistant phenotype. Therefore given the range of CTX-M production in these isolates a subset of 23 isolates were tested to determine the impact CTX-M β-lactamase production had on piperacillin/tazobactam and ceftolozane/tazobactam zone size. Disc diffusion breakpoints for ceftolozane/tazobactam are currently set by the FDA and available for only *Pseudomonas aeruginosa*; therefore, susceptibility data for this drug combination for *E. coli* could not be determined. Only zone size comparisons could be made. In spite of the high-level expression from the CTX-M-15 gene, 7 of 15 isolates were susceptible to the piperacillin/tazobactam. Isolates W15 and H15 were resistant to piperacillin/tazobactam, while the remaining isolates were intermediate (Table 1). All of the CTX-M-14-producing isolates listed in Table 1 were susceptible to piperacillin/tazobactam. With respect to ceftolozane/tazobactam, all the isolates listed in Table 1 regardless of the level of protein production observed had zone sizes of ≥19 mm. An additional 126 isolates not evaluated for protein production, but possessing either CTX-M-14 or CTX-M-15 β-lactamases, were also tested against ceftolozane/tazobactam. All 50 of the CTX-M-14-like-producing isolates had zone diameters for ceftolozane/tazobactam of ≥21 mm. Of the 76 additional isolates producing CTX-M-15-like enzymes, 73 showed zone diameters to ceftolozane/tazobactam of ≥19 mm, while 2 of 76 isolates had zone diameters of 18 mm and 1 of 76 had a zone diameter of 14 mm.

## Discussion

Previous studies evaluating the effectiveness of ceftolozane/tazobactam against isolates that produced CTX-M-14 or CTX-M-15 indicated that the concentration of tazobactam may play a role in the overall efficacy of this combination when used against these ESBL-producing bacteria.^32,33^ When a breakpoint of 1 mg/L of ceftolozane plus 4 mg/L of tazobactam was used, 12% of isolates evaluated by Titelman *et al*.^33^ were non-susceptible. A similar finding for ESBL-producing Enterobacteriaceae and ceftolozane/tazobactam has been reported by Livermore *et al*.^32^ It is possible that the level of CTX-M production was responsible for the lack of susceptibility to ceftolozane/tazobactam in these isolates. Given our observation that CTX-M-15 isolates in general transcribed more mRNA than CTX-M-14 isolates, we wanted to evaluate the impact that this discrepancy in mRNA expression would have on CTX-M protein production and ultimately susceptibility to β-lactam/β-lactamase inhibitor combinations. To our surprise, the level of CTX-M protein production for most of the isolates evaluated was similar for CTX-M-15 and CTX-M-14 producers despite the differences in mRNA expression. Therefore, the level of CTX-M production was most likely not responsible for the resistance observed in the 12% of isolates evaluated by Titelman.^33^ The choice of breakpoint for ESBL-producing bacteria is controversial^34^ and EUCAST and CLSI have set lower breakpoints for oxyimino-cephalosporins to increase the probability of classifying isolates as non-susceptible. Our data show that the variation in susceptibility in ESBL producers for ceftolozane/tazobactam is not due to levels of β-lactamase production, so this criterion for establishing breakpoints maybe an oversimplification.

The evaluation of the CTX-M-14 and CTX-M-15 mRNA half-lives indicated that the prolonged CTX-M-15 half-life observed for many of the CTX-M-15 producers was controlled by a factor encoded on the CTX-M-15-harbouring plasmid(s). This conclusion is substantiated by several experiments. First, transconjugants from CTX-M-14- or 15-producing donor strains had mRNA half-lives equivalent to the corresponding donor strains (Table 3). Second, transformants of cloned CTX-M-14 and CTX-M-15 genes into identical plasmid vectors had *bla*_CTX-M_ mRNA half-lives of <2 min regardless of the genetic background, including a ST131 *E. coli* (FHM16). Third, native or heterologous promoters expressing *bla*_CTX-M_ alleles did not influence the mRNA half-life. These data also indicated that the plasmid backbones of CTX-M-15-encoding isolates were not identical since some of these isolates had mRNA half-lives of ≤2 min, whereas others had half-lives of ≥5 min. We speculate that the CTX-M-15-encoding plasmids that exhibited shorter half-lives have a similar composition to the CTX-M-14-encoding plasmids with respect to the encoded factor that determines mRNA half-life.

Steady-state levels of mRNA expression in the transconjugants indicated that for two of the three CTX-M-15-producing isolates (XQ12 and C15) evaluated, the genetic background did not influence promoter usage, plasmid copy number or transcript half-life. However, the steady-state level of mRNA for the CTX-M-15-producing XQ35 transconjugants in the FHM16 and MG1655 backgrounds was 4–6.5-fold higher compared with *bla*_CTX-M-15_ expression in the XQ35 clinical isolate. It is possible that when *bla*_CTX-M-15_ was expressed in the FHM16 and K12 MG1655 backgrounds, the copy number of the plasmid or transcription initiation events were altered compared with expression in the XQ35 and J53 backgrounds. Copy number has been shown to change even when the same plasmid vector is used, but transformed into different hosts (genetic backgrounds) of Enterobacteriaceae.^35^

Sequence analysis of CTX-M-14/CTX-M-15-containing plasmids has shown multiple open reading frames of hypothetical proteins that could encode for a factor that influences the CTX-M transcript half-life.^36–38^ A protein that can modify mRNA half-life and is encoded by a plasmid has multiple implications. This factor could not only prolong the half-life of antibiotic resistance gene transcripts, as demonstrated in this study, but also influence the production of factors responsible for virulence or metabolic pathways, leading to increased fitness for strains that carry *bla*_CTX-M-15_. Identification of this factor should be a priority, since it could serve as a potential target for the development of new antimicrobial agents to limit the production of proteins benefiting from a prolonged mRNA half-life.

The ability to evaluate β-lactamases in clinical isolates using hydrolysis data is difficult for isolates that carry multiple β-lactamases as in the ones evaluated in this study. Therefore, to evaluate the level of protein production of CTX-M β-lactamases only, immunoblot analysis was used. The use of an internal control to normalize the data is difficult using clinical isolates, as expression of any internal housekeeping protein may fluctuate in response to various selective pressures faced by a given organism. Therefore, the use of Stain-Free technology allows for a more accurate comparison of relative amounts of protein among clinical isolates.^29^

The difference observed between the levels of CTX-M-15 mRNA and protein suggests that these isolates are inefficient in translating all the transcribed mRNA into functional β-lactamase. Although some of the mRNA is translated, as evident from the immunoblot data, the level of CTX-M-15 β-lactamase did not result in decreased zone sizes to either tested inhibitor combination. This suggests that the rate of penetration of antibiotics through the outer membrane may be important when evaluating CTX-M-producing resistant isolates. Even though there are no disc diffusion breakpoints for Enterobacteriaceae currently available for ceftolozane/tazobactam, comparisons between MIC data and zone size suggest that a zone size of ≥19 mm could be considered susceptible (Cubist data on file). However, those data still contain some major errors between some isolates regarding MIC versus disc zone size that need to be resolved (Cubist data on file). None the less, most of the isolates in this study (126 of 129) had zone sizes to ceftolozane/tazobactam of ≥19 mm despite the elevated levels of protein production observed. Notably, isolate RS135 produced the highest level of mRNA and the second highest level of CTX-M-15 protein, yet showed a zone size of 19 mm for ceftolozane/tazobactam. In contrast, the highest protein production was noted for strain JJ2246F, but the ceftolozane/tazobactam zone of inhibition was 30 mm indicating that the level of β-lactamase did not contribute to smaller zone sizes for these isolates.

It was surprising that the levels of mRNA expression observed for most CTX-M-15-producing isolates were disproportional to protein production. This disproportional correlation between mRNA and protein production could be the result of a translational block due to an RNA binding protein or small RNA interference, or the result of decreased CTX-M-15 protein stability compared with CTX-M-14. The differential levels of expression between mRNA and protein production did not seem to be influenced by phylogenetic background since no correlations could be determined with ST or phylotype. As the selective pressure increases with β-lactam/β-lactamase inhibitor combinations or carbapenems, a future concern is the selection of genomic or plasmidic mutations that will allow increased translatability of the CTX-M transcripts resulting in increased β-lactamase production. These events could lead to an emergence of CTX-M-producing organisms resistant to β-lactam/β-lactamase inhibitor combinations and may increase the number of isolates resistant to the carbapenems through porin down-regulation. Fortunately, our current findings suggest that resistance in *E. coli* to β-lactam/β-lactamase inhibitor combinations including ceftolozane/tazobactam is not dependent on the type or level of CTX-M β-lactamase produced by the *E. coli* isolate, but requires as yet unidentified resistance mechanisms.

## Funding

This study was supported in part by a grant from Merck Inc., and STRECK.

## Transparency declarations

J. R. J. has received consultancies, grants and/or contracts from ICET, Janssen, Merck, Syntiron and Tetraphase. S. J. W. has received grant salary support from the Pfizer Medical Education Committee and is party to a patent for a rapid molecular test to inform antibiotic selection in the treatment of urinary tract infection due to *E. coli*, but to date has received no stock options or any royalties for this technology. P. H. has received in the last 3 years lecture/consultancy fees from Eumedica, Merck and Pfizer, and research funding from Novartis and Pfizer. N. D. H. serves as a consultant for Cubist Pharmaceuticals. All other authors: none to declare.

## Supplementary data

Tables S1 and S2 and Figures S1 to S7 are available as Supplementary data at *JAC* Online (http://jac.oxfordjournals.org/).

Supplementary Data
