# Supplementary material for: Evaluation of CTX-M steady-state mRNA, mRNA half-life and protein production in various STs of Escherichia coli
Source: J Antimicrob Chemother. 2015 Nov 26;71(3):607–16. doi: 10.1093/jac/dkv388 (PMC4743699; doi:10.1093/jac/dkv388)
Supplement: Supplementary Data [file supp_71_3_607__index.html]

Evaluation of CTX-M steady-state mRNA, mRNA half-life and protein production in various STs of Escherichia coli — Evaluation of CTX-M steady-state mRNA, mRNA half-life and protein production in various STs of Escherichia coli — Supplementary Data 

# Evaluation of CTX-M steady-state mRNA, mRNA half-life and protein production in various STs of *Escherichia coli*

## Supplementary Data

Supplementary Data

- Supplementary Data - Docx file
