# Supplementary material for: Evaluation of CTX-M steady-state mRNA, mRNA half-life and protein production in various STs of Escherichia coli
Source: J Antimicrob Chemother. 2015 Nov 26;71(3):607–16. doi: 10.1093/jac/dkv388 (PMC4743699; doi:10.1093/jac/dkv388)
Supplement: Supplementary Data [file supp_dkv388_dkv388supp.docx]

**Supplementary data**

**Table S1.** Plasmid vectors used in this study

| **Plasmid** | **Copy Number in *E. coli*** | **Characteristics** | **Source** |
| --- | --- | --- | --- |
| pCR^®^2.1 | 100-200 | kan^R^, amp^R^, pUC ori | Invitrogen Life Technologies |
| pJET1.2/blunt |  | amp^R^, pMB1ori | Fermentas, Thermo Scientific |
| pACYC184 | 15 | tet^R^, cat^R^, p15A1 ori | ATCC |
| MDR009 | 15 | tet^R^, cat^S^, p15A1 ori | 9 |
| pMP220 | 12 | tet^R^, promoter-less | Herbert Schweizer laboratory |
| pUCP26 | ND | tet^R^, pBR322 ori | Herbert Schweizer laboratory |

Table S2. Primers used for this study

| **Primer Name** | **Sequence (5′ to 3′)** | **Nucleotide Position** | **GenBank Accession Number^a^** | **Purpose** |
| --- | --- | --- | --- | --- |
| **CTX-M-14** | | | | |
| CTXM-914F | GCTGGAGAAAAGCAGCGGAG | 1857-1876 | AF252622 | Sequencing |
| CTXM-914R | GTAAGCTGACGCAACGTCTG | 2330-2311 | AF252622 | Sequencing |
| CTXM14-R1 | GCCTGAATAGCTTCATCATCC | 2799-2779 | AF252622 | Sequencing/Cloning |
| CTXM14-RTF1 | GCTCAAAGGCAATACGACC | 2379-2397 | AF252622 | Expression, ½ life |
| CTXM14-RTR1 | GCTGGGTAAAATAGGTCACC | 2545-2526 | AF252622 | Expression, ½ life |
| **CTX-M-15** | | | | |
| CTX3-FLF | cgtatcttccagaataaggaatccc | 1957-1981 | HQ157357.1 | Sequencing |
| CTX3-FLR | GTTTCCCCATTCCGTTTCCGC | 2880-2860 | HQ157357.1 | Sequencing/Cloning |
| CTXM-1F3 | gacgatgtcactggctgagcttagc | 2335-2359 | HQ157357.1 | Sequencing |
| CTXM-1R2 | AGCCGCCGACGCTAATACA | 2833-2815 | HQ157357.1 | Sequencing |
| CTXM15-RTF1 | CCGTCACGCTGTTGTTAGG | 2028-2046 | HQ157357.1 | Expression, ½ life |
| CTXM15-RTR1 | CCATCACTTTACTGGTGCTGC | 2216-2196 | HQ157357.1 | Expression, ½ life |
| **Others** | | | | |
| ISEcp1 site2 F2 | gtaacaaatactaccttgctttctg | 1141-1165 | AF252622 | Sequencing/Cloning |
| ISEcp1 site3 F3 | gcggacctagattctacgtcag | 38-59 | AF252622 | Sequencing |
| Frr-F1 | TGATCGTTCAATGTCTCCG | 210-228 | EU906107 | Expression |
| Frr-R1 | GCTTGTTCTGCTTCACCAC | 371-353 | EU906107 | Expression |
| 16srRNAEcKp-F1 | GAGAGGATGACCAGCCACAC | 212-231 | JX975437.1 | Expression, ½ life |
| 16srRNAEcKp-R1 | CGCCCATTGTGCAATATTCC | 295-276 | JX975437.1 | Expression, ½ life |
| **Luciferase-fusion clones** | | | | |
| KpnISEcp1-UF1 | GGGTACCGAAGGTTCCGAATACGAC | 1111-1128 | AF252622 | Cloning |
| HindCTX14-UR1 | AAAGCTTCTCAAACTCCCAATACGG | 1740-1723 | AF252622 | Cloning |
| HindCTX15-UR1 | AAAGCTTGGGATTCCTTATTCTGGAAG | 1981-1962 | HQ157357.1 | Cloning |
| XbaLucR1 | CTCTAGAATTACACGGCGATCTTTCC | 1698-1680 | U47122 | Cloning |
| **Heterologous promoter clones** | | | | |
| CTXM14-lacF1 | GGGATCCGAGATGGTGACAAAGAGAGTGC | 1738-1759 | AF252622 | Cloning |
| CTXM14-lacR1 | AAAGCTTCCAGTTACAGCCCTTCGG | 2620-2603 | AF252622 | Cloning |
| CTXM15-lacF1 | GGGATCCCATGGTTAAAAAATCACTGCG | 1981-2001 | HQ157357.1 | Cloning |
| CTXM15-lacR1 | AAAGCTTCTATTACAAACCGTCGGTGAC | 2860-2840 | HQ157357.1 | Cloning |
| CTXM14-EcoF1 | GAGATGGTGACAAAGAGAGTGC | 1738-1759 | AF252622 | Cloning |
| CTXM14-EcoR1 | CCAGTTACAGCCCTTCGG | 2620-2603 | AF252622 | Cloning |
| CTXM15-EcoF1 | CATGGTTAAAAAATCACTGCG | 1981-2001 | HQ157357.1 | Cloning |
| CTXM15-EcoR1 | CTATTACAAACCGTCGGTGAC | 2860-2840 | HQ157357.1 | Cloning |

|  |  |  |  |
| --- | --- | --- | --- |

a; Sequence used to design primer

**Linear range response experiments**

The linear response range of the Stain-Free fluorescence and the anti-CTX-M antibody for bacterial lysates was determined by performing a Western blot on a dilution series of total protein ranging from 40 μg to 0.625 μg as previously described (OpdQ manuscript). The Stain-Free signal was compared to the amount of protein loaded per lane and linear regression was used to assess its linearity (Figure S1). Second, the linearity of the anti-CTX-M antibody chemiluminescence signal was evaluated using the anti-CTX-M antibody (1:45,000) and a secondary anti-rabbit IgG antibody (1:50,000). The chemiluminescence detected from the secondary antibody bound to the anti-CTX-M antibody was compared to the amount of protein loaded per lane and linear regression was used to assess the linearity of the anti-CTX-M chemiluminescence signal (Figure S2). The resulting Stain-Free signal demonstrated linearity ranging from 5 μg to 40 μg and the linear range of the anti-CTX-M antibody was from 2.5 μg to 40 μg. Since the Stain-Free and chemiluminescent signals have linear responses in the range of protein tested, the amount of CTX-M in the cell could be accurately detected and compared among bacterial isolates.

**Figure S1.**

**Figure S2.** Evaluation of the linear range of the anti-CTX-M antibody using a dilution series of total protein from D14 *E. coli* ranging from 40 μg to 0.625 μg.

**Figure S3.** Half-life graphs for the CTX-M-15-producing clinical isolate XQ12 and its transconjugants.

**Figure S4.** Half-life graphs for the CTX-M-15-producing clinical isolate C15 and its transconjugants.

**Figure S5.** Half-life graphs for the CTX-M-15-producing clinical isolate XQ35 and its transconjugants.

**Figure S6.** Half-life graphs for the CTX-M-14-producing clinical isolates D14 and XQ10 and their transconjugants.

**Figure S7.** Half-life graphs for the CTX-M-14- and CTX-M-15-producing transformants.
